# Protein Structural Model Selection by Combining Consensus and Single Scoring Methods

**DOI:** 10.1371/journal.pone.0074006

**Published:** 2013-09-02

**Authors:** Zhiquan He, Meshari Alazmi, Jingfen Zhang, Dong Xu

**Affiliations:** Department of Computer Science and Christopher S. Bond Life Sciences Center, University of Missouri, Missouri, United States of America; University of Michigan, United States of America

## Abstract

Quality assessment (QA) for predicted protein structural models is an important and challenging research problem in protein structure prediction. Consensus Global Distance Test (CGDT) methods assess each decoy (predicted structural model) based on its structural similarity to all others in a decoy set and has been proved to work well when good decoys are in a majority cluster. Scoring functions evaluate each single decoy based on its structural properties. Both methods have their merits and limitations. In this paper, we present a novel method called PWCom, which consists of two neural networks sequentially to combine CGDT and single model scoring methods such as RW, DDFire and OPUS-Ca. Specifically, for every pair of decoys, the difference of the corresponding feature vectors is input to the first neural network which enables one to predict whether the decoy-pair are significantly different in terms of their GDT scores to the native. If yes, the second neural network is used to decide which one of the two is closer to the native structure. The quality score for each decoy in the pool is based on the number of winning times during the pairwise comparisons. Test results on three benchmark datasets from different model generation methods showed that PWCom significantly improves over consensus GDT and single scoring methods. The QA server (MUFOLD-Server) applying this method in CASP 10 QA category was ranked the second place in terms of Pearson and Spearman correlation performance.

## Introduction

Protein three-dimensional (3D) structures are of great significance for protein function analysis. Experimental methods for protein structure determination such as X-ray crystallography and nuclear magnetic resonance (NMR) are costly and time consuming. Computational prediction provides an economic way to bridge the increasing gap between the number of available protein primary sequences and 3D structures. It is also a viable and efficient approach to study proteins [1]. For example, high-accuracy models can be directly used in studying catalytic activity of enzymes and provide a basis for drug design. Structural models with medium accuracy up to 6 Å of Root-Mean-Square Deviation (RMSD) are often useful for understanding protein functions [2]. Although efforts of decades, protein structure prediction from primary sequences is still a research challenge [3], [4].

Most recent protein structure prediction methods such as Rosetta [5], I-TASSER [6], [7], [8] and MUFOLD [9] adopt a sampling procedure in which quite a number of candidate models (decoys) are generated. One of the remaining issues is how to select good models that are close to the native structure. Quality assessment (QA) for protein structure models is therefore highly important. Although a lot of work has been done, it still remains as one of the bottlenecks in practical predictions and has large room to improve.

QA methods roughly fall into two categories. The first one is a single model assessment approach, which takes one single structure as input and assigns a score to indicate its structural similarity or distance to the native. In this category, physical-based energies [10], [11] calculate atomic level energies of a decoy according to physical principles. However, the energy value is sensitive to minor changes in structure, and hence it is hard to apply it to QA. Knowledge-based scoring functions rely on statistical distributions of atoms in native structures. For example, OPUS-Ca [12] uses the distance distributions of residue pairs and DDFire [13] constructs residue-specific all-atom potential of mean force from a database of native structures. Several methods based on sequence-structure relationship train a scoring function or machine-learning model to estimate the quality of the predicted models [14], [15], [16], [17], [18]. For example, QMean [18] combines sequence-structure information such as atom-atom contact, secondary structure prediction and solvent accessibility into a scoring function to predict the quality of single models. The second category of QA methods take a set of structures as input and use the information from the decoy set to assign a score to each structure member. The most widely used method is the consensus approach, such as naïve Consensus Global Distance Test (CGDT) [19] which assigns a score to each decoy as its average structural similarity (GDT score) to all other members in the set. This works well when good models are among a major cluster, especially in CASP (Critical Assessment of Protein Structure Prediction) [20], where all participating groups try to submit their best models. MUFOLD-WQA [21] is a variation of pure consensus approach which introduces a weight for each pair of decoys. Consensus and knowledge-based scoring functions reveal different but complementary aspects of structural models. The consensus method utilizes geometric information exclusively from the decoy set without taking advantage of the biophysical or statistical properties within and between primary sequences and 3D structures. Several works have been done to combine the two approaches using machine-learning methods such as neural network (NN) or support vector machine (SVM) [18], [22], [23], [24], [25]. Noticing the fact that knowledge-based scoring functions are relatively “noisy” and have low correlations to the actual decoy quality, in [26] we developed a protein-dependent scoring method to combine consensus and single scoring functions for decoy selection. In this method, the optimal weights for each of the component scores were obtained in the first optimization step to increase their discerning power. Then in the second step, the weighted scores and other sequence-related features were directly mapped to the GDT score of each decoy by an SVM. This method achieved some improvement over CGDT and single scoring functions in selection performance. Almost all the combination methods mentioned above use machine learning modules to capture the complex and remote relationship between feature scores and the decoy structural quality, such as GDT score. And the performance still has large room to improve. In this paper, we proposed a new method to combine consensus GDT and knowledge-based scoring functions to obtain better QA performance. First, a consensus score called Position Specific Probability Sum (PSPS) was developed as one of the features. Here, the structural state of each residue in a decoy was represented by the bond angles of four consecutive residues. Thus, each decoy was represented by a sequence of structure codes (states). A probability score was calculated for each decoy of a set based on consensus. Although this method alone did not have outstanding performance in decoy selection, it was quite different from all other methods, and outperformed CGDT when combined with other methods such as OPUS-Ca [12], DDFire [13] and RW [27]. Second, a two-stage method was developed to perform QA. We trained two neural-network models to sequentially capture the underlying correlation among different features (scoring functions). Specifically, for every two decoys, the first neural-network model decided whether they were structurally close or not in terms of their GDT scores to the native, and subsequently, the second model determined which one was closer to the native. After the comparison between all pairs of decoys, we calculated a score for each decoy in the pool based on the number of winning times. We applied this method to three benchmark data sets from different protein structure prediction methods and demonstrated significant improvements over CGDT and state-of-art single scoring functions in terms of best model selection performance and Spearman correlation to actual GDT score. We also modified this method to attend the QA category of CASP 10 in 2012 and the server (MUFOLD-Server) was ranked the second place in terms of Pearson and Spearman correlation performance.

## Methods

### Position Specific Probability Sum (PSPS) Score

**Figure 1:**
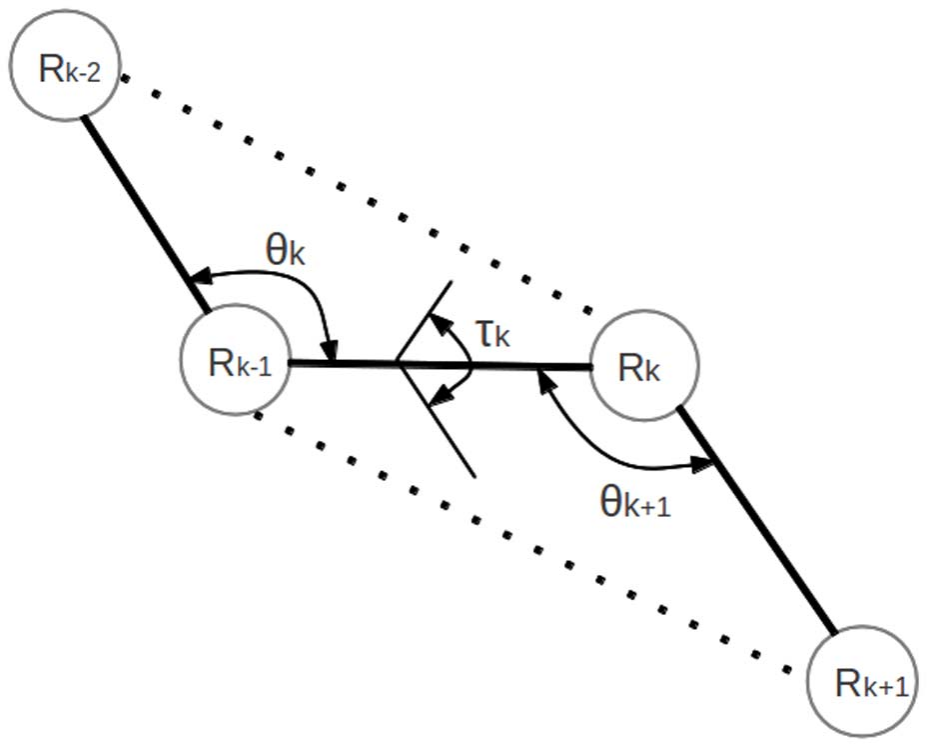
Angle representation of four consecutive residues.



To obtain PSPS score, each decoy in a decoy set was transformed to a sequence of structural code based on the study in [28]. For each residue 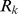, we calculated an angle triplet 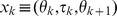 for four consecutive C-alpha atoms, where 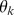 is the bend angle of 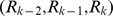, 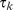 is the dihedral angle of 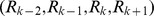 and 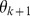 is the bend angle of 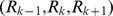, as shown in Figure 1. 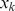 is assigned to one of the 17 clusters (states) according to the following Gaussian Mixture Model:

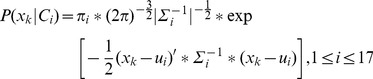
(1)

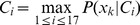
(2)


After we had all the structure-code sequences of the decoys for the same protein, we calculated Position Specific Frequency Matrix (PSFM). 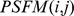 is the occurring frequency of state 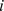 at sequence position 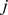, where 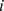 is the state index from 1 to 17, representing the 17 clusters; 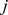 is the residue position from *3* to *L-1* (*L* is the length of protein). This matrix was counted in the decoy pool and normalized by dividing the number of decoys. We then got Position Specific Probability Sum (PSPS):
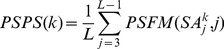
(3)where 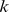 is the decoy index and 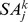 is the cluster state of position 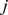 in the structure code (state) sequence of decoy 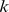, which is calculated by Eqn. 2.

### Combine Consensus and Single Scoring Functions

For a set of decoys of a target protein, the input features for every decoy-pair were the respective differences between OPUS-Ca, RW, DDFire, PSPS and CGDT of the two decoys.
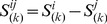
(4)where 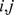 are decoy indexes, and 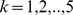 represents five different scores.

Two neural-network models were used to compare a decoy pair. Model 1 was trained to determine whether two decoys were significantly different or not in terms of the GDT scores to their native. We chose the cutoff to be 0.025, which meant that if the GDT difference of two decoys was larger than 0.025, they were treated as being significantly different. Model 2 was used to predict whether one decoy was better than the other. To train this model, considering the training error, we removed those of pairs wherein the GDT difference was less than 0.01 from training data. Model 2 was tested only on the pairs that were predicted to be significantly different by Model 1. Both classifying neural networks had the same configuration, which is one hidden layer of 3 nodes with sigmoid activation functions, and same input feature vectors of five dimensions, each of which is specified by Eqn. 4. After the comparison between all pairs of decoys, the final score, named as PWCom, for each decoy was simply the number of winning times during the pair-wise comparisons. The training and testing were done in a leave-one-out manner at protein (target) level, which meant each target (decoy set) was tested on the models trained on all other targets (decoy sets).

### Datasets

**Figure 2:**
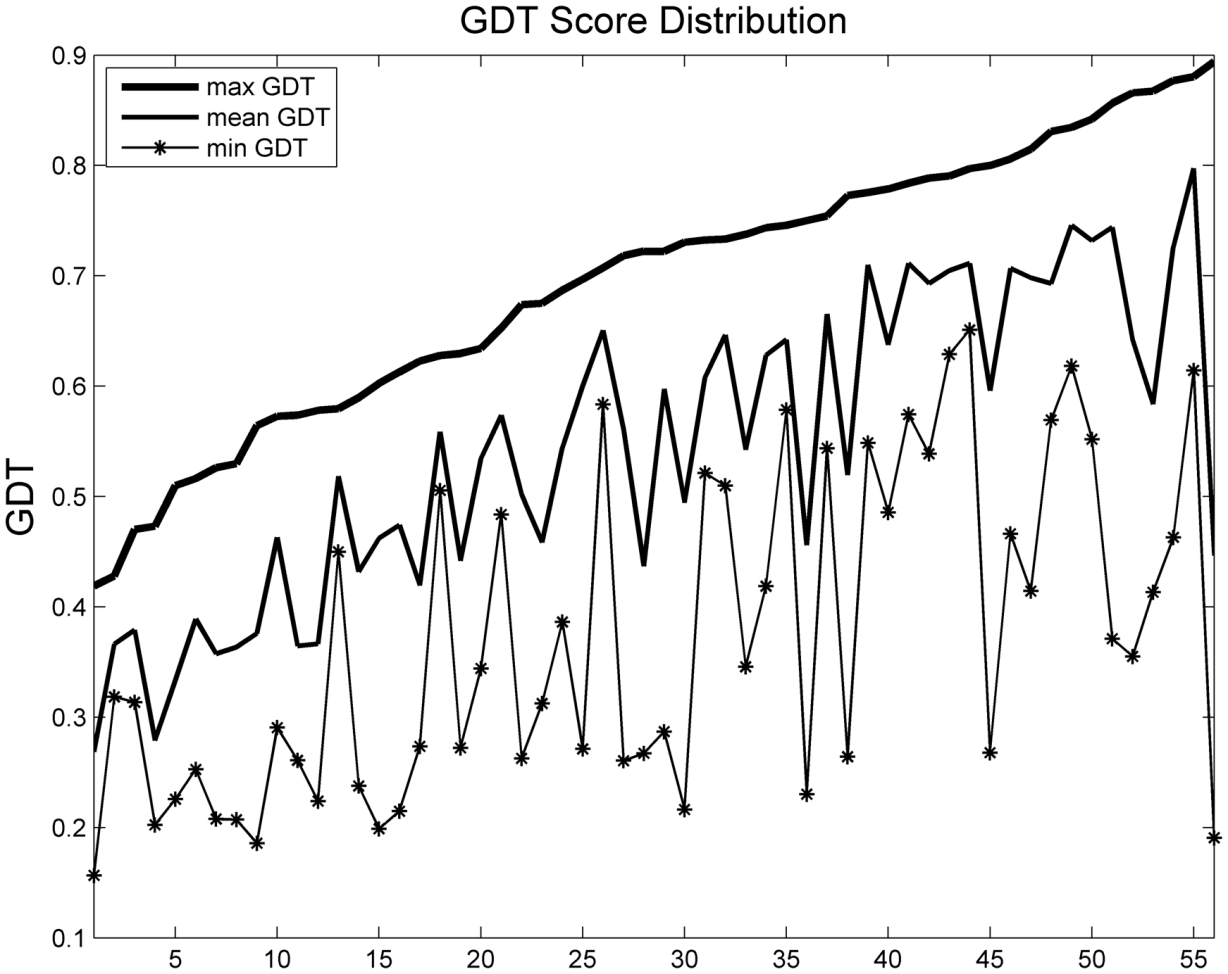
GDT score distribution of benchmark 1.

**Figure 3:**
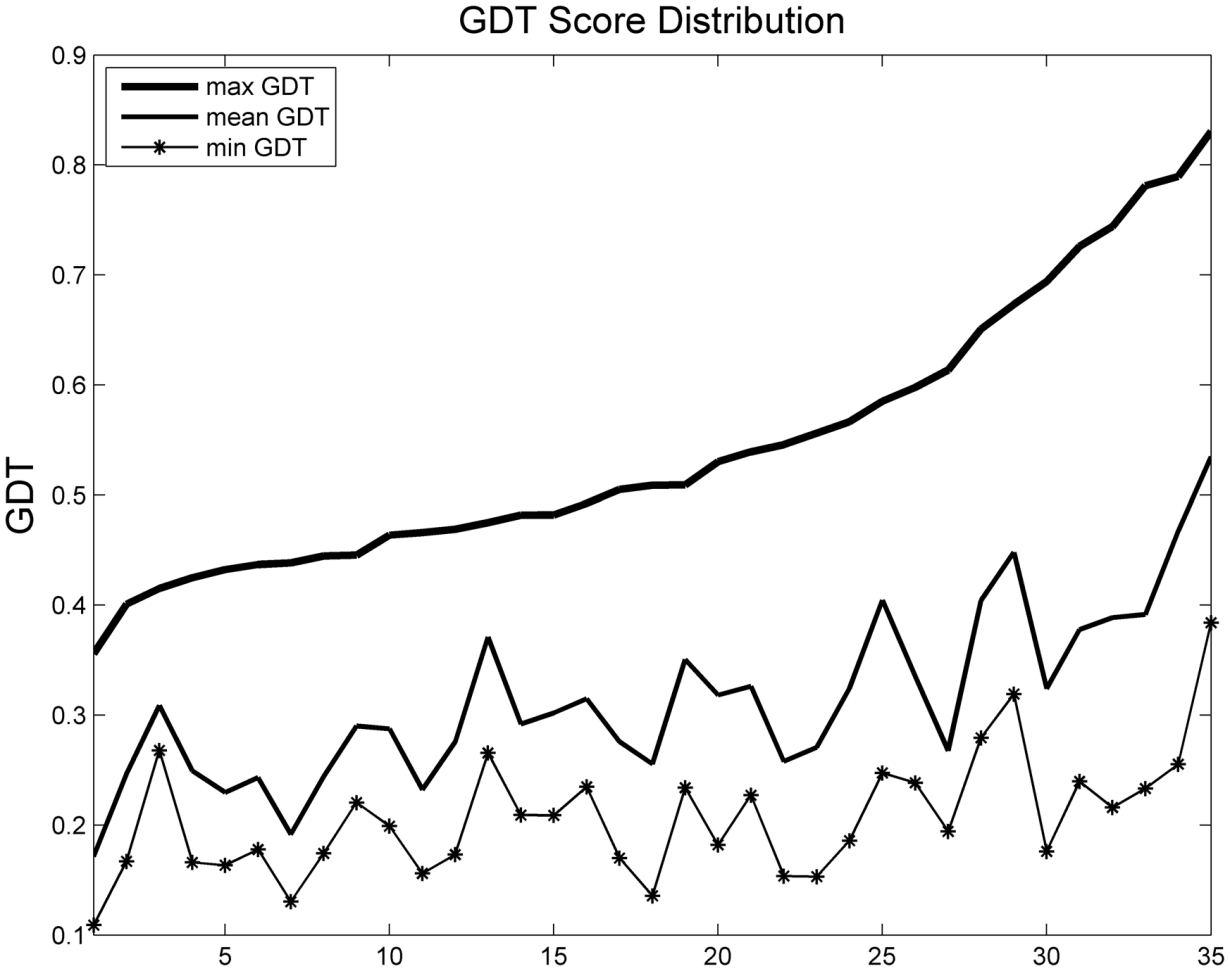
GDT score distribution of benchmark 2.

**Figure 4:**
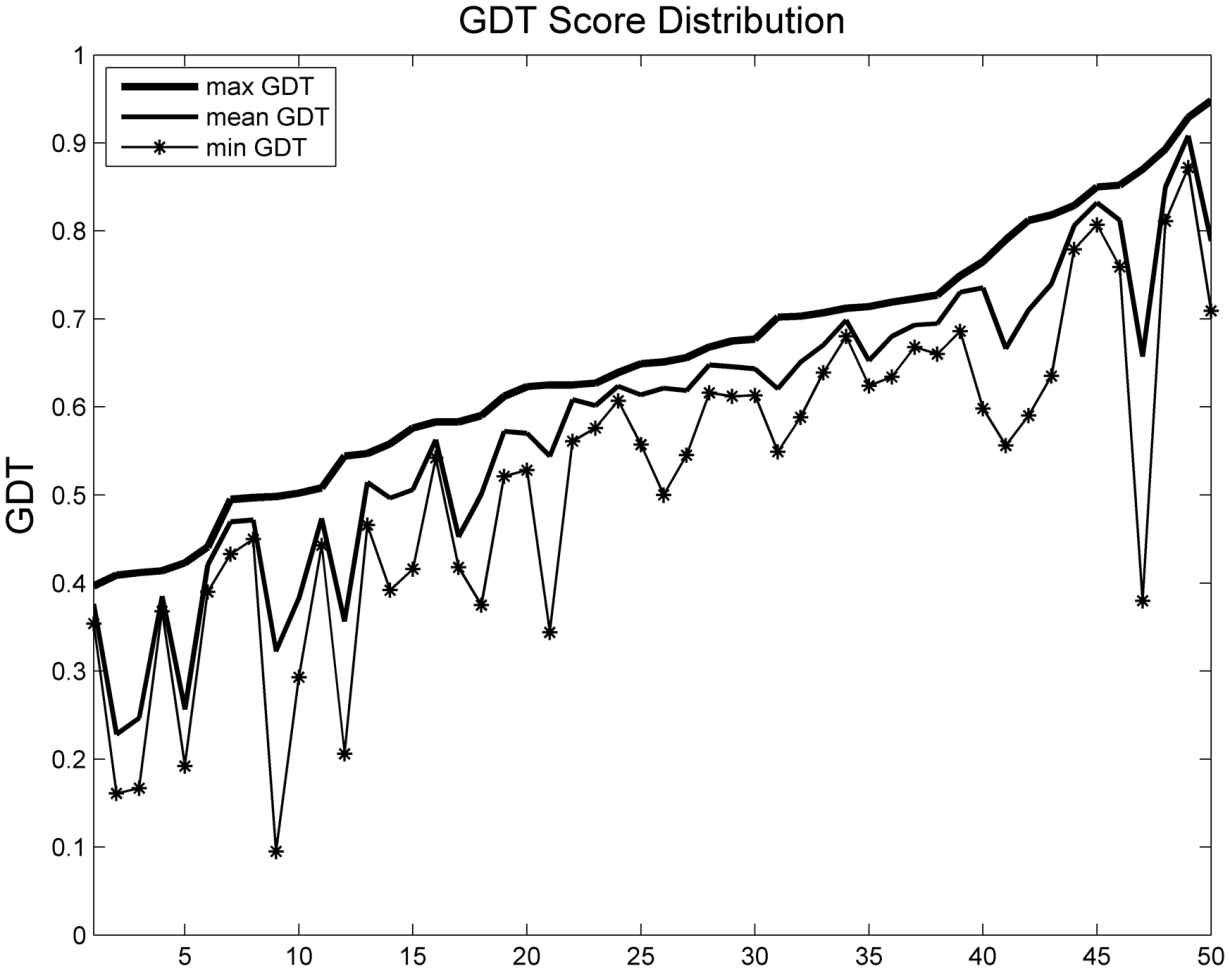
GDT score distribution of benchmark 3.

We applied this method to three benchmark datasets from different model prediction methods. Each target (protein) had hundreds of decoys. The best decoy in each target had a GDT score greater than 0.4, which ensured that the pool contained reasonably good decoys. The first dataset contained 56 targets with decoys generated by I-TASSER *ab initio* modeling method (http://zhanglab.ccmb.med.umich.edu/decoys/) [7]. The second dataset consisted of 35 CASP 8 [20] targets predicted by Rosetta or Robetta. The third dataset contained 50 CASP 9 targets with decoys generated by our in-house template-based model generation tool MUFOLD. Figures 2, 3, and 4 show the GDT distribution information, i.e., maximum, average and minimum GDT of each dataset respectively.

### Modification for CASP 10 QA Category

We applied this method to attend the QA Section 1 in CASP 10 worldwide competition in 2012. In this QA section, 150 CASP server models were selected using naïve consensus GDT method for each protein. Usually, for these subsets of decoys, applying a naïve consensus GDT method again does not work well. Considering the fact that some of decoys in CASP are incomplete, which affects the performance of scoring functions like OPUS-Ca, DDFire and RW, we used another set of feature scores in the following:

1. Secondary structure match score between the predicted secondary structures from sequence by PSIPRED [29] and the actual secondary structures calculated from decoy by DSSP [30].
2. Solvent accessibility match score between the predicted solvent accessibility from sequence by SSPro [31] and the actual solvent accessibility calculated by DSSP.
3. Mean square error between predicted backbone 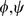 dihedral angles by SPINE [32] and actual dihedral angles from decoys by DSSP.
4. Structural environment fitness score which measures the propensity of an amino acid type to appear in a structural environment specified by secondary structure type and solvent accessibility type [26].
5. Naïve Consensus GDT score, CGDT.

Our automated CASP 10 QA server was trained using CASP 9 decoy data sets.

## Results

In the test, each score was used to rank the decoys of a given protein. We studied the selection performance using three measures to compare each method. In the following comparison tables, “GDT1” is the average GDT score of the top-1 model; “avgGDT5” is the average of the mean GDT score of the top 5 models; “Pearson” is the average Pearson correlation coefficient to actual GDT score and “Spearman” is the average Spearman correlation coefficient to actual GDT score. As Spearman is the correlation between the respective ranks given by two scores instead of the actual scores values, we use Spearman as the major correlation coefficient to measure protein structure selection performance.

### Performance Statistics

As shown in Tables 1, 2 and 3, CGDT has better performance than all other single scoring functions in terms of the three measures. Specifically, in benchmark 1, although CGDT’s top-1 selection performance is not significantly better than that of other feature scores, its correlation (Spearman: 0.5845) is much higher than the others, among which DDFire is the best (Spearman: 0.4403). In benchmark 2, CGDT is significantly better than OPUS-Ca, DDFire, RW and PSPS in terms of all three measures. Its top-1 selection performance (average GDT: 0.4255) has more than 3 GDT points than DDFire (0.3901), which is the best among the remaining feature scores. In benchmark 3, the top-1 selection performance of all feature scores are similar, but in terms of Spearman correlation, CGDT is still the best (0.3199) with DDFire in second place (0.3049).

**Table 1:** Performance on benchmark 1.

	Benchmark 1		
	GDT1	avgGDT5	Spearman
GDT	0.6946	0.6767	1.0000
CGDT	0.6058	0.6039	0.5845
DDFire	0.6006	0.5906	0.4403
OPUS-Ca	0.5959	0.5925	0.4156
RW	0.5954	0.5879	0.4172
PSPS	0.5847	0.5734	0.3299
PWCom	**0.6105**	**0.6056**	**0.6011**
WQA [26]	0.6098	0.6034	

**Table 2:** Performance on benchmark 2.

	Benchmark 2		
	GDT1	avgGDT5	Spearman
GDT	0.5449	0.5219	1.0000
CGDT	0.4255	0.4060	0.5584
DDFire	0.3901	0.3788	0.2722
OPUS-Ca	0.3763	0.3663	0.2739
RW	0.3662	0.3696	0.2766
PSPS	0.3435	0.3534	0.2462
PWCom	**0.4529**	**0.4309**	**0.5615**
WQA [26]	0.4446	0.4220	

**Table 3:** Performance on benchmark 3.

	Benchmark 3		
	GDT1	avgGDT5	Spearman
GDT	0.6503	0.6431	1.0000
CGDT	0.6023	0.6042	0.3199
DDFire	0.6091	0.6094	0.3049
OPUS-Ca	0.6054	0.6085	0.2395
RW	0.6008	0.6056	0.2233
PSPS	0.5987	0.6002	0.2307
PWCom	**0.6131**	**0.6136**	**0.3377**

From Tables 1, 2 and 3, we can see that PWCom is significantly and consistently better than CGDT in three benchmarks. Notably, in benchmark 2, the top GDT performance of PWCom is much higher than that of CGDT, with the improvement of 0.4529–0.4255 = 0.0274. In the other two benchmarks, PWCom score still improves in average top-1 GDT performance over CGDT, and even more over single scoring functions. As for Spearman correlation, PWCom is consistently better than CGDT in all three benchmarks.

### Case Study

**Figure 5:**
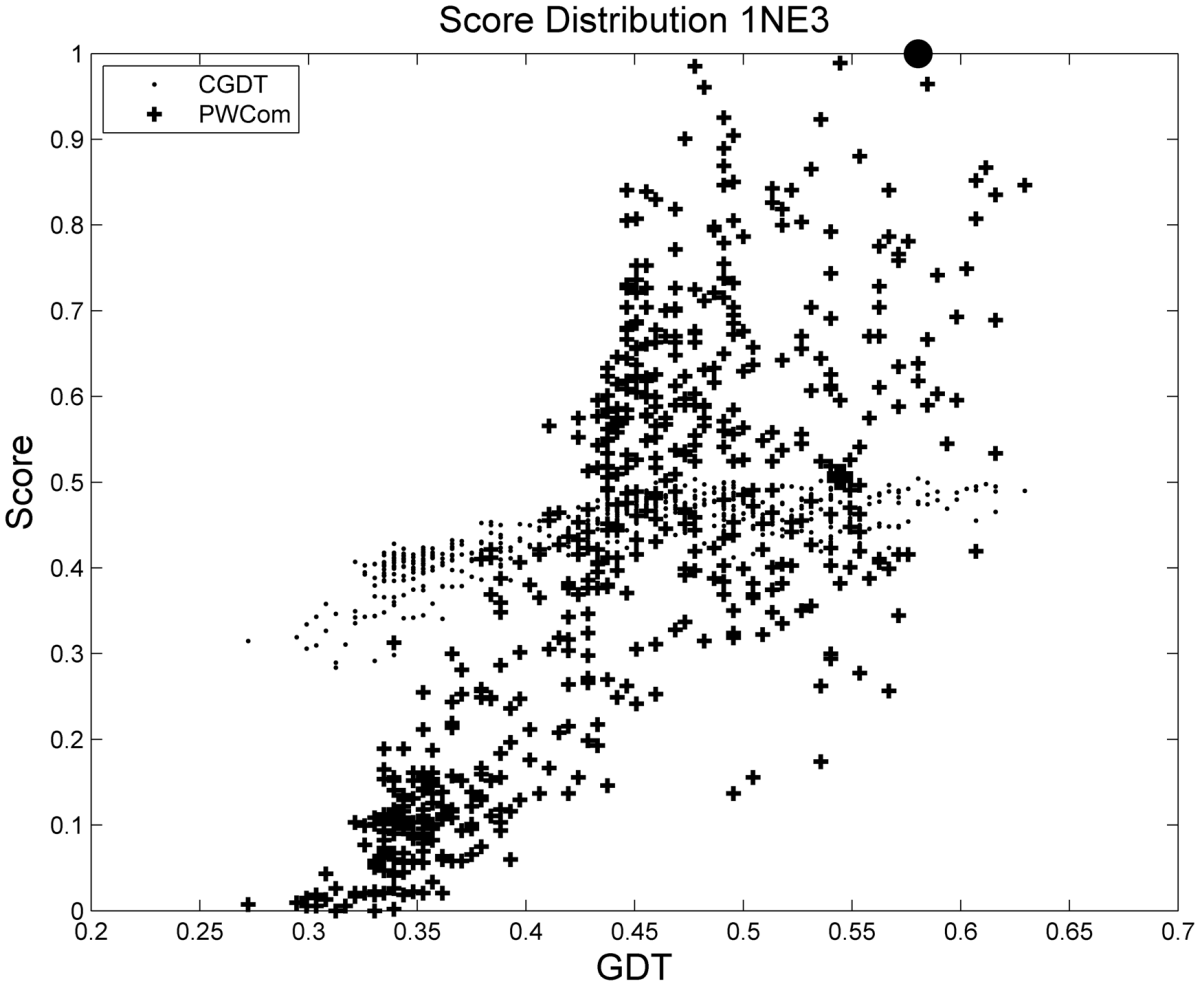
Distribution of CGDT and PWCom for 1NE3 from benchmark 1. The black circle on the top is the selected best decoy according to PWCom and the black box at the bottom according to CGDT.

In addition to average performance shown above, we use some individual cases from these benchmark datasets to see more detailed comparisons. Figure 5 and Table 4 shows target 1NE3 from benchmark 1. Figure 5 compares the distribution of CGDT and PWCom for this decoy set, and Table 4 shows the QA performance. We can see that in this target, CGDT has a higher Spearman correlation coefficient than that of OPUS-Ca, RW, DDFire and PSPS, but its top-1 or top-5 selection performance is not the best. PWCom achieves the best performance in top-1 selection performance with similar performance to CGDT in Spearman correlation.

**Table 4:** Comparison of 1NE3 from benchmark 1.

	GDT1	avgGDT5	Spearman
GDT	0.6295	0.6179	1.0000
CGDT	0.5446	0.5268	0.7551
DDFire	0.4464	0.4696	0.2675
OPUS-Ca	0.5670	0.5330	0.3870
RW	0.5134	**0.5411**	0.1437
PSPS	0.4330	0.5035	0.1739
PWCom	**0.5804**	0.5339	**0.7556**

**Figure 6:**
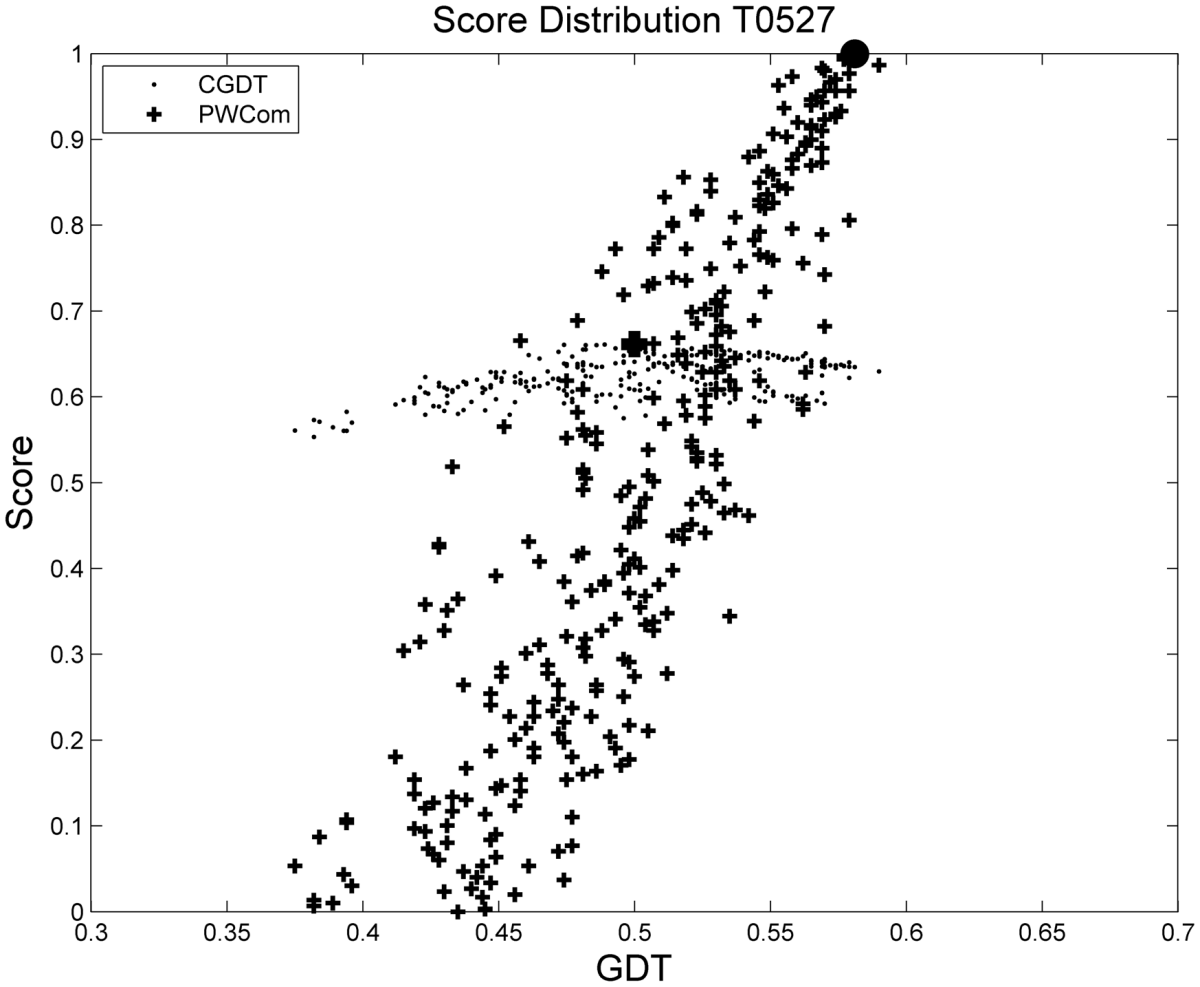
Distribution of CGDT and PWCom for T0527 from benchmark 3. The black circle on the top is the selected best decoy according to PWCom and the black box at the bottom according to CGDT.

Figure 6 and Table 5 shows target T0527 from benchmark 3. For this target, most of the single scoring functions are better than CGDT; for example, the Spearman correlation coefficient of CGDT is only 0.3924, while the one of OPUS-Ca is as high as 0.8886. PWCom combines these scores to achieve the best selection performance. From Figure 6, we also see that PWCom has much better correlation with GDT than that of CGDT.

**Table 5:** Comparison of T0527 from benchmark 3.

	GDT1	avgGDT5	Spearman
GDT	0.5900	0.5816	1.0000
CGDT	0.5000	0.4896	0.3924
DDFire	0.5770	0.5808	0.8157
OPUS-Ca	0.5670	0.5670	**0.8886**
RW	0.5770	0.5744	0.7709
PSPS	0.5350	0.5150	0.3013
PWCom	**0.5810**	**0.5808**	0.8742

**Figure 7:**
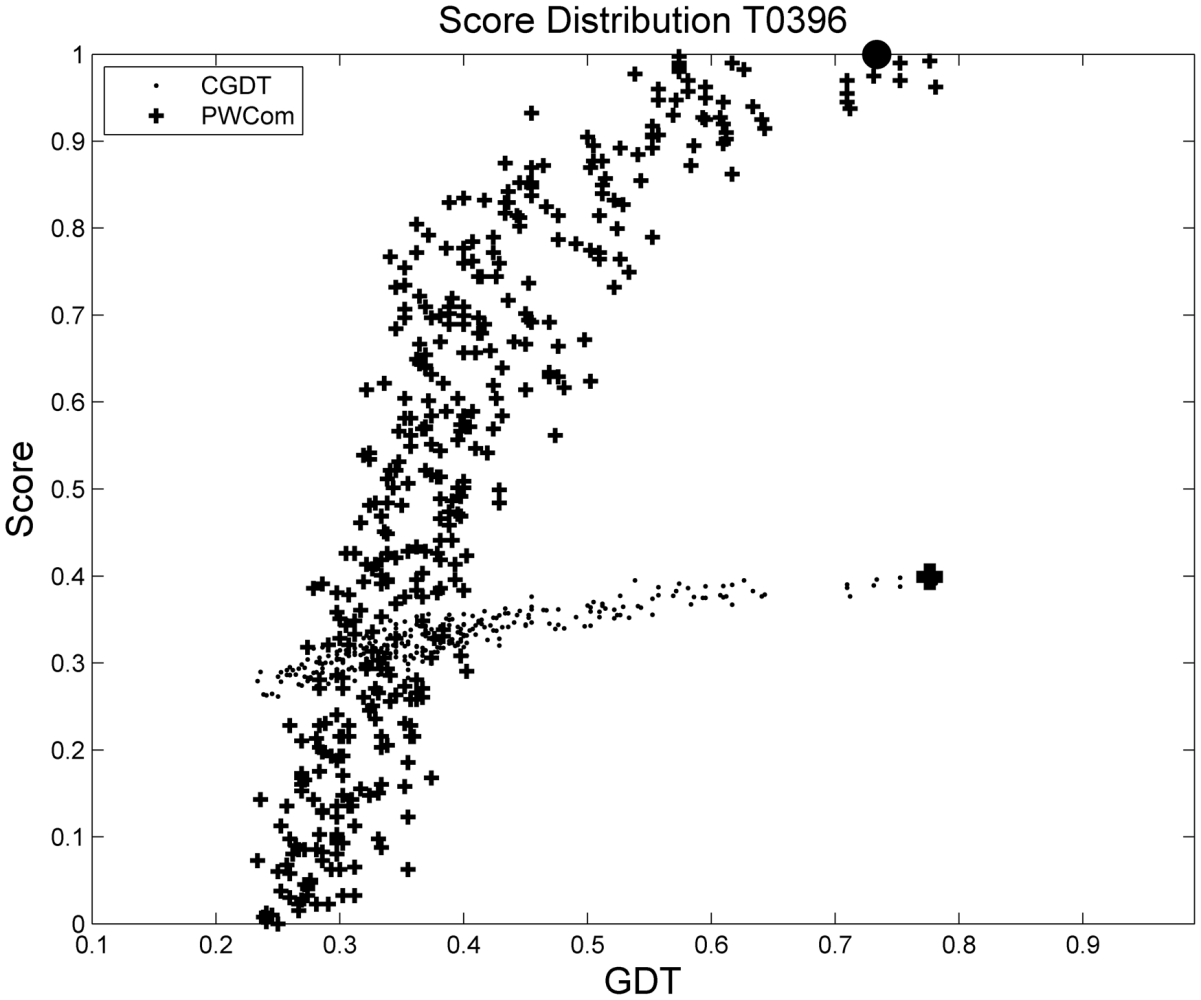
Distribution of CGDT and PWCom for T0396 from benchmark 2. The black circle on the top is the selected best decoy according to PWCom and the black box at the bottom according to CGDT.

Figure 7 and Table 6 show target T0396 from benchmark 2. In this case, CGDT is the best performer in terms of all three measures. From Figure 7, we can see that CGDT is almost linearly correlated with the actual GDT scores and Table 6 shows CGDT selects nearly the best one in the decoy set. Although PWCom has better performance than single scoring functions, it does not improve over GDT in this case. This might be because CGDT is already very good as shown in the big performance gap between CGDT and single scoring functions, and there is no room to further improve it in this particular case.

**Table 6:** Comparison of T0396 from benchmark 2.

	GDT1	avgGDT5	Spearman
GDT	0.7810	0.7591	1.0000
CGDT	**0.7762**	**0.6852**	**0.9098**
DDFire	0.4000	0.4724	0.3677
OPUS-Ca	0.2595	0.4586	0.1514
RW	0.3786	0.4029	0.2600
PSPS	0.6071	0.5919	0.4990
PWCom	0.7333	0.6819	0.8954

### Performance in CASP 10

Table 7 shows the top 15 out of total 37 servers in CASP10 QA section 1 in terms of Pearson and Spearman correlation. The MUFOLD-Server was ranked at the second best server in both Pearson and Spearman performance. Especially, its total Spearman score was 52.612, which was nearly the same as the best server performance (52.766).

**Table 7:** Performance in CASP 10.

Server	Pearson correlation (X100)	Spearman correlation (X100)
Pcomb	52.921	52.766
**MUFOLD-Server**	**51.486**	**52.612**
Pcons	46.389	46.579
ProQ2clust	50.11	50.002
MULTICOM-CONSTRUCT	49.621	49.797
ConQ	45.707	46.333
MULTICOM-REFINE	49.796	50.116
PconsQ	45.668	45.97
GOAPQA	47.431	51.843
MQAPsingle	45.703	45.868
MQAPfrag	45.703	45.868
MQAPfrag2	45.703	45.868
MQAPmulti	45.897	45.801
ModFOLDclust2	47.556	47.699
Ariadne	43.227	46.669

## Discussion

Our new approach combines the advantages of consensus GDT method and single scoring functions through pairwise comparison and a two-stage machine-learning scheme. Consensus GDT method depends on the decoy distribution and relies on geometric information of protein structures only, while single scoring functions produce a wide range of values for different decoys, which makes their scores unstable and noisy. Our method tries to capture the correlation between score differences and actual structural quality difference as well as the complementarity among these scores. It does so through a pairwise comparison between any two decoys. Although this approach takes more computing time than ranking single scores directly, it is more sensitive to capture the differences among models and less prone to systematic errors of single scores on the decoys. Because of using single score information, PWCom is more correlated to the real GDT score with respect to the native structure than consensus methods.

Our test result shows that PWCom was better than CGDT or single scoring functions in selection performances (GDT1 or avgGDT5) and correlations. PWCom was also better than the previous WQA method [26]. This may be because WQA trained a SVM to directly map feature scores like CGDT, OPUS-Ca score etc. to actual GDT scores of decoys, which is less stable generally when applied to different kinds of structural models. In addition, the weights of WQA for single scoring functions were optimized through quadratic programming, which required much more computation than PWCom.

PWCom’s performance is affected by the performances of the feature scores, and hence may vary in individual cases. For example, in the target shown in Figure 7 and Table 6, PWCom is worse than CGDT. Like CGDT and WQA, PWCom was also inevitably affected by the decoy distribution. Comparing Table 2 to Tables 1 and 3, we can see PWCom score got more improvement over other scores in benchmark 2 than those in benchmarks 1 and 3. For example, in benchmark 2, the top-1 selection performance of PWCom was 0.4529, while the best of others was CGDT (0.4255). The improvement (0.4529–0.4255) was 0.0274; while in benchmarks 1 and 3, the improvement was less significant. Comparing the decoy distributions of benchmark 2 to 1 and 3, the gap between maximum and mean GDT curve in Figure 3 was much bigger than that of Figures 2 and 4 [26]. Furthermore, for quite a few targets in benchmarks 1 and 3, the gap between maximum and minimum GDT was quite small. This may explain why the average top-1 selection performance of single scoring functions was close to that of CGDT in these two benchmarks. In spite of this, in terms of Spearman correlation, CGDT was still better than single scoring functions.

One important factor when combining different scoring methods is the correlation or redundancy within these methods. In [33], before linear combination of the predictions from different methods, principal component analysis was carried out to reduce the correlations among them. Highly correlated scores are not good for our combination, although neural networks are more tolerant of feature redundancy and have more power to capture complex relationships. Table 8 shows the average Spearman correlation among the scores on benchmark 3. As we can see, among the 5 feature scores, RW and DDFire have the highest average correlation coefficient, which is 0.698 The correlation between CGDT and PSPS is 0.652, which means that RW and DDFire, as well as CGDT and PSPS may encode similar information. We tested the procedure on benchmark 3 with each component score removed at a time. Table 9 shows the performance of each test. As it shows, the correlation performance of PWCom decreases dramatically after removing RW or DDFire, although they have the highest average correlation. Removing PSPS does not result in as much decreases as other tests. However, it is not obvious to see the relationship between the features and the final QA performance. The best way is to test different feature scores and study their correlations, which may lead to more significant QA improvements. Another issue is the significant similarity among different structural models due to related prediction methods, which has been addressed in previous studies [16]. We plan to improve our method using such an approach in the future.

**Table 8:** Average spearman correlations between scores on benchmark 3.

	GDT	CGDT	DDFire	OPUS-Ca	RW	PSPS	PWCom
GDT	1.000	0.320	0.305	0.240	0.223	0.231	0.338
CGDT		1.000	0.380	0.340	0.288	**0.652**	0.630
DDFire			1.000	0.420	**0.698**	0.438	0.702
OPUS-Ca				1.000	0.359	0.400	0.702
RW					1.000	0.296	0.386
PSPS						1.000	0.635
PWCom							1.000

**Table 9:** Performance on benchmark 3 with 4 features.

	GDT1	avgGDT5	Pearson	Spearman
PWCom	0.6131	0.6136	0.3328	0.3377
PWCom, No CGDT	0.6081	0.6062	0.2928	0.2971
PWCom, No PSPS	0.6141	0.6102	0.3243	0.3320
PWCom, No DDFire	0.6071	0.6081	0.2688	0.2713
PWCom, No RW	0.6022	0.6054	0.2624	0.2647
PWCom, No OPUS-Ca	0.6031	0.6053	0.3034	0.3071

There are other aspects that this method can be improved in terms of training errors and parameter optimization. We empirically chose 0.025 as the cutoff for neural-network model 1 and 0.01 to screen training data for neural-network model 2. Large-scale training and testing may help find better values for these cutoffs and other parameters in this approach. On the other hand, in terms of model training itself, we trained two neural-network models to predict whether two decoys are similar and which one is better than the other. An alternative machine learning method, such as regression, might help further improve over classification methods. Once these issues are addressed and the method is fine-tuned, we plan to release PWCom to the public.
